# Supplementary material for: The Effects of Fucoidan Derived from Sargassum filipendula and Fucus vesiculosus on the Survival and Mineralisation of Osteogenic Progenitors
Source: Int J Mol Sci. 2024 Feb 8;25(4):2085. doi: 10.3390/ijms25042085 (PMC10889223; doi:10.3390/ijms25042085)
Supplement: Supplementary file 1 [file ijms-25-02085-s001.zip › ijms-2815917-supplementary.pdf]

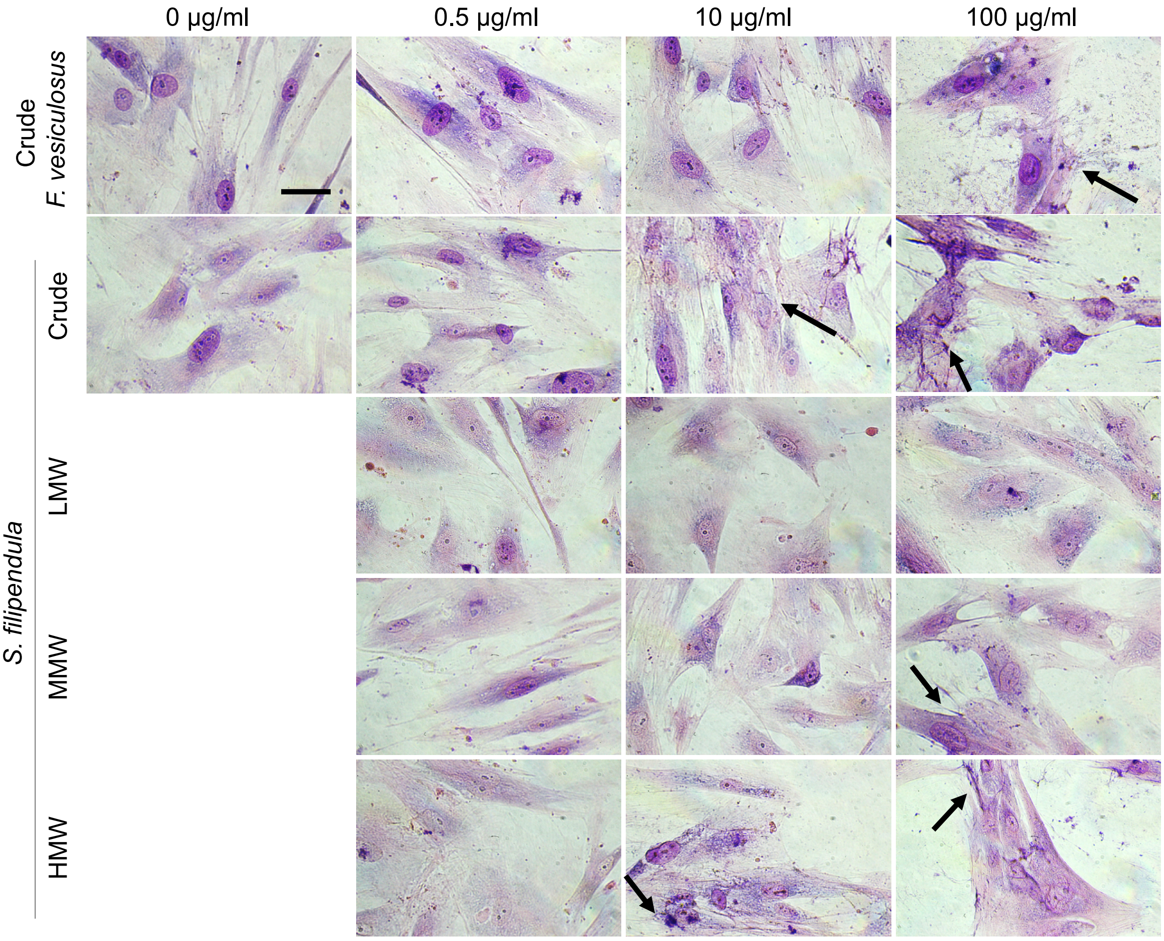

Supplementary Figure S1: Giemsa stained hES-MPs cells after 5 days treatment with different fucoidans. Scale bar- 100 µm. Arrows- distorted cell morphology and cell debris.

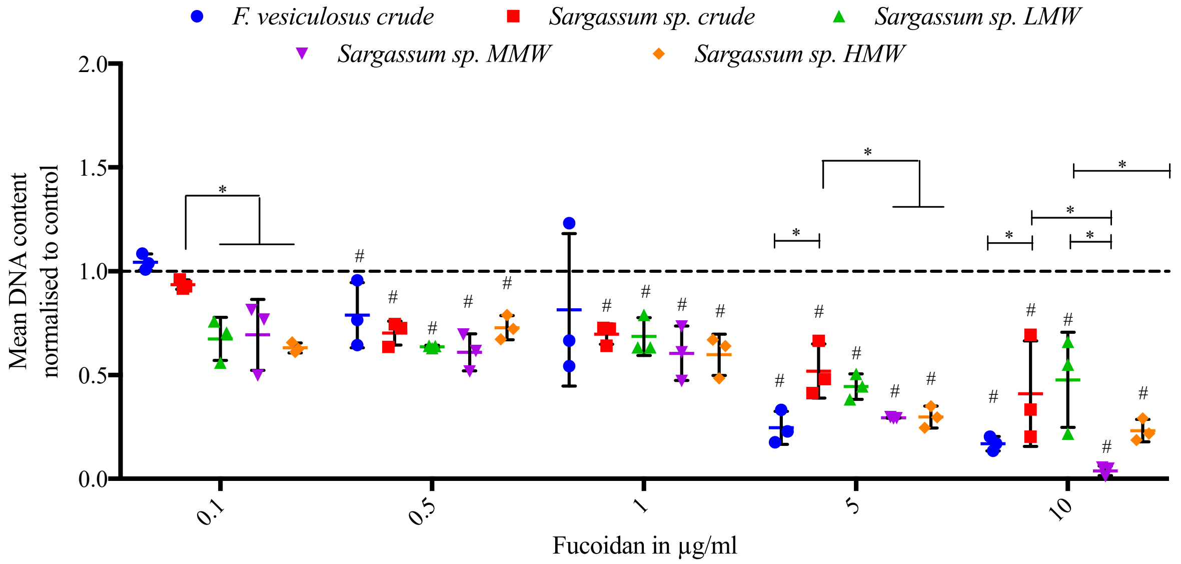

Supplementary Figure S2: Assessment of DNA content (n = 3) of hES-MPs after 21 days of treatment with different fucoidans from 0-10 µg/ml. ---- indicates vehicle control (0 µg/ml). * $p < 0.05$, # $p < 0.05$ relative to respective vehicle control.

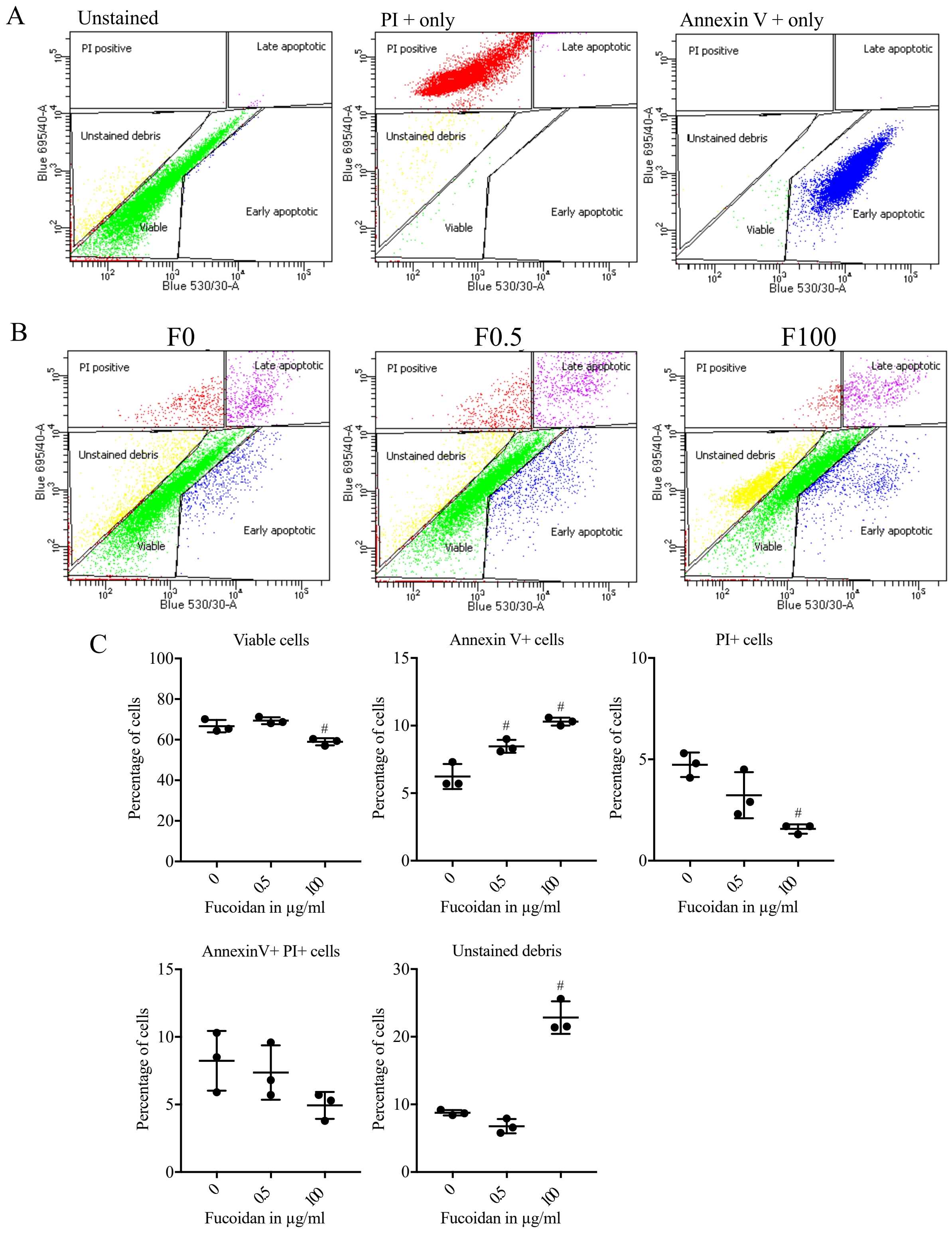

Supplementary Figure S3: Apoptotic analysis for hES-MPs treated with 0, 0.5 and 100 µg/ml of crude fucoidan extract derived from *F. vesiculosus*. (A) Gating strategy using unstained, PI+ only and Annexin+ only cells. (B) Cells were seeded at 20,000 cells/cm^2 and next day fucoidan treatment was started lasting 10 days. (C) Mean ± S.E.M. of different cell populations, n = 3. Notice the presence of significantly higher amounts

of unstained debris in case of 100 µg /ml fucoidan dose. # $p < 0.05$ relative to respective vehicle control.
